# IDO1 is highly expressed in macrophages of patients in advanced tumour stages of oral squamous cell carcinoma

**DOI:** 10.1007/s00432-022-04277-7

**Published:** 2022-08-13

**Authors:** Ann-Kristin Struckmeier, Anne Radermacher, Michael Fehrenz, Tamara Bellin, Dalia Alansary, Philipp Wartenberg, Ulrich Boehm, Mathias Wagner, Anja Scheller, Jochen Hess, Julius Moratin, Christian Freudlsperger, Jürgen Hoffmann, Lorenz Thurner, Klaus Roemer, Kolja Freier, Dominik Horn

**Affiliations:** 1 Department of Oral and Maxillofacial Surgery, Saarland University Medical Center, Kirrberger Str. 100, 66421 Homburg, Saar Germany; 2 Institute of Biophysics, Center for Integrative Physiology and Molecular Medicine (CIPMM), Saarland University, Homburg, Saar Germany; 3 Department of Experimental and Clinical Pharmacology and Toxicology, Center for Molecular Signaling (PZMS), Saarland University, Homburg, Saar Germany; 4 Department of Pathology, Saarland University Medical Center, Homburg, Saar Germany; 5 Department of Molecular Physiology, Center for Integrative Physiology and Molecular Medicine (CIPMM), Saarland University, Homburg, Saar Germany; 6 Department of Otorhinolaryngology, Head and Neck Surgery, University Hospital Heidelberg, and German Cancer Research Center (DKFZ), Heidelberg, Germany; 7 Department of Oral and Maxillofacial Surgery, University Hospital Heidelberg, Heidelberg, Germany; 8 Department of Internal Medicine 1 (Oncology, Hematology, Clinical Immunology, and Rheumatology), Saarland University Medical Center, Homburg, Saar Germany; 9 José Carreras Center for Immuno and Gene Therapy, Saarland University, Homburg, Saar Germany

**Keywords:** IDO, Oral squamous cell carcinoma (OSCC), Macrophage, Tumour microenvironment, Immunotherapy

## Abstract

**Purpose:**

Strategies for Indolamine-2,3-dioxygenase 1 (IDO1) inhibition in cancer immunotherapy once produced encouraging results, but failed in clinical trials. Recent evidence indicates that immune cells in the tumour microenvironment, especially macrophages, contribute to immune dysregulation and therefore might play a critical role in drug resistance.

**Methods:**

In this study, we investigated the significance of IDO1 expressing immune cells in primary tumours and corresponding lymph node metastases (LNMs) in oral squamous cell carcinoma (OSCC) by immunohistochemistry. The link between IDO1 and macrophages was investigated by flow cytometry in tumour tissue, healthy adjacent tissue and peripheral blood mononuclear cells (PBMCs). IDO1 activity (measured as Kynurenine/Tryptophan ratio) was assessed by ELISAs.

**Results:**

High IDO1 expression in tumour-infiltrating immune cells was significantly correlated with advanced stages [Spearman’s rank correlation (SRC), *p* = 0.027] and reduced progression-free survival (multivariate Cox regression, *p* = 0.034). IDO1 was significantly higher expressed in PBMCs of patients in advanced stages than in healthy controls (ANOVA, *p* < 0.05) and IDO1^+^ macrophages were more abundant in intratumoural areas than peritumoural (*t* test, *p* < 0.001). IDO1 expression in PBMCs was significantly correlated with IDO1 activity in serum (SRC, *p* < 0.05). IDO1 activity was significantly higher in patients with LNMs (*t* test, *p* < 0.01).

**Conclusion:**

All in all, IDO1 expressing immune cells, especially macrophages, are more abundant in advanced stages of OSCC and are associated with reduced progression-free survival. Further investigations are needed to explore their role in local and systemic immune response. The IDO1 activity might be a suitable biomarker of metastasis in OSCC patients.

**Supplementary Information:**

The online version contains supplementary material available at 10.1007/s00432-022-04277-7.

## Introduction

Oral squamous cell carcinoma (OSCC) is the most common type of head and neck squamous cell carcinoma (HNSCC) with an annual incidence of more than 300.000 new cases worldwide (Bray et al. 2017).

Currently available treatment approaches are surgical resection, radiotherapy, chemotherapy, and immunotherapy. Unfortunately, the 5-year overall survival rate has remained at approximately 50% for the last decades (Blatt et al. 2017). Therefore, designing new effective strategies and seeking specific targets in cancer immunotherapy is of great importance.

Indoleamine-2,3-dioxygenase 1 (IDO1) is an enzyme catabolizing the first and rate-limiting step of the Kynurenine (Kyn) pathway and is expressed in epithelial cells, macrophages and dendritic cells (Mbongue et al. 2015; Mellor and Munn, 2004; Munn et al. 1999). IDO1 is involved in various processes exerting immunosuppressive effects [e.g. prevention of foetal rejection during pregnancy (Mellor et al. 2001), suppression of autoimmune disease (Scott et al. 2009)].

In the past, several IDO1 inhibitors were evaluated as possible new anticancer drugs (Yao et al. 2021). Initial trials showed promising therapeutic effects, but phase 3 trials were finally terminated, because they could not increase the efficacy of treatment, while showing more side effects (Long et al. 2019).

Recent evidence indicates that the tumour microenvironment (TME) mainly attributes to drug resistance (Ruffell and Coussens 2015). Especially the tumour associated macrophages (TAMs) attracted the interest of basic and clinical scientists, since they are abundant in the TME and were suggested to play a crucial role in tumour proliferation, tumour maintenance, angiogenesis, and metastasis (Colegio et al. 2014; Pan et al. 2020; Valastyan and Weinberg, 2011). Thereby, understanding the immunosuppressive local TME and the systemic immunological environment are critically important for translational cancer research.

According to Mills, TAMs can be classified according to their activation state into the M1 phenotype (antitumour, classically activated) and the M2 phenotype (pro-tumour, alternatively activated; Mills et al. 2000). The subpopulations of macrophages coexist within tumours and the understanding of their relationship, their functions and their intra- and intertumoural differences are potential keys to improve current cancer therapies.

Epithelial cancer cells have been shown to express IDO1 (Laimer et al. 2011), but little is known about its expression in immune cells. Since IDO1 is involved in the first and rate-limiting step of the catabolism of Tryptophan (Trp) to Kyn, the Kyn/Trp ratio is suggested to reflect IDO1 activity (Badawy and Guillemin 2019).

The aim of this study was to determine the significance of IDO1 expressing immune cells in OSCC with a focus on macrophages. In the retrospective part of this study, we investigated the IDO1 expression in immune cells in primary tumours (PTs) and corresponding lymph node metastases (LNMs) in a homogenous cohort of OSCC patients. Secondly, we investigated the significance of IDO1^+^ macrophages in peripheral blood mononuclear cells (PBMCs) and compared their abundance between tumour tissue and healthy adjacent tissue prospectively. Moreover, we investigated the levels of Kyn and Trp in serum as surrogate for IDO1 activity to examine the suitability of IDO1 activity as potential biomarker for cancer stage and therapy response. In the final step, we investigated the correlation between IDO1 activity in serum with IDO1 expression in PBMCs and epithelial cancer cells.

## Materials and methods

### Patients

Immunohistochemistry (IHC) was performed on tissue microarrays (TMAs) to determine IDO1 expression in PTs and LNMs. The OSCC patients received diagnosis and primary surgical treatment including tumour resection and neck dissection at the Department of Oral and Maxillofacial Surgery at the University Hospital Heidelberg according to the national guideline for OSCC therapy between 2010 and 2016.

The prospective cohort for flow cytometric analysis and enzyme-linked immunosorbent assay (ELISA) was recruited at the Department of Oral and Maxillofacial Surgery at Saarland University Medical Center between 2020 and 2022. Blood samples were collected at time of the primary surgical treatment including tumour resection and neck dissection as well as 1 and 2 weeks after surgery. Moreover, tumour and healthy adjacent tissues were collected at the tumour resection. Additionally, blood samples of healthy controls (HCs) were collected. None of the patients enrolled had received previous chemotherapy or radiotherapy, nor did they have a history of prior malignancies. IDO1 expression might be influenced by several diseases, thereby patients with dementia, depression, diabetes mellitus, and autoimmune diseases were excluded for the HC group.

Written informed consent was obtained from all patients before their involvement in the study. The study design and methods were approved by the Ethics Committees of the Heidelberg University (Ethic vote: S-360/2011) and the University of Saarland (Ethic vote: 37/20).

The histopathological diagnosis and the differentiation grade of the tumours were provided from the Departments of Pathology at University Hospital Heidelberg and Saarland University Medical Center. The surgical margins of the removed tissue were negative in all patients.

### Immunohistochemistry

IDO1 expression in 171 PTs and 40 corresponding LNMs was investigated by IHC. TMAs were prepared following published protocols (Freier et al. 2003; Moratin et al. 2019).

Slides were stained anti-IDO1 monoclonal antibody (clone D5J4E, Cell Signaling Technology, Cambridge, United Kingdom) at a dilution of 1:400 following the manufacturer’s instructions and were scanned with an Axio Scan.Z1 (Zeiss, Oberkochen, Germany) for further investigation. The TMAs were scored using ZEN (blue edition) program (Zeiss) by three independent observers.

TMAs were assessed by determining the density of IDO1^+^ immune cells and distinguishing the distribution of IDO1^+^ immune cells between peritumoural and intratumoural regions.

We used a dataset previously published by Moratin et al. to evaluate the correlation between IDO1 and PD-L1/2 (Moratin et al. 2019). In situ hybridization for human papillomavirus (HPV)-DNA was performed following published protocols (Kühn et al. 2021; Linxweiler et al. 2015). Immunohistochemical staining targeting p16 was performed following the manufacturer’s instructions (clone EPR1473, Abcam, Cambridge, United Kingdom).

### Blood samples

Venous blood was obtained from 19 tumour patients and 4 volunteers for HCs. The serum was clotted for 10 min at room temperature (RT) prior to centrifugation at 1500*g* for 5 min. The serum was collected into tubes and placed in Mr. Frosty (Nalgene, NY, USA) overnight at − 80 °C. The next day, tubes were transferred to nitrogen.

### Peripheral blood mononuclear cell preparation

PBMCs were isolated by centrifugation using a Ficoll density gradient. First, blood samples were diluted 1:1 with PBS (Thermo Fisher Scientific, MA, USA) and thoroughly mixed. Next, 20 ml of Ficoll-Paque media (Ficoll-Paque Plus, GE Healthcare, IL, USA) was poured at the bottom of a 50 ml conical tube and the cell suspension was carefully layered on the top. Tubes were centrifuged at 1750*g* for 20 min at RT without applying any brake. The layer of mononuclear cells was transferred to a 50 ml conical tube. Cells were washed twice using 10 ml of PBS each time. The amount and the viability of the cells were assessed by the Luna-FX7 Automated Cell Counter (Logos Biosystems, Anyang-si, South Korea). Subsequently, cells (2 × 10^6^ cells per tube) were resuspended in 90% FBS and 10% DMSO. Tubes were placed in Mr. Frosty (Nalgene) overnight at − 80 °C. The next day, tubes were transferred to nitrogen.

For thawing, cells were placed in a 37 °C water bath and then transferred to pre-warmed RPMI (20 ml). Cells were washed twice using RPMI.

### Preparation of single cell suspension

We obtained fresh tumour samples from 9 patients with primary OSCC. Moreover, we obtained healthy adjacent tissues from 6 of these patients. Tissue samples were stored in MACS Tissue Storage Solution and transferred to laboratory immediately. Tissues were dissociated using the Tumour Dissociation Kit human in combination with the gentleMACS Octo Dissociator with heaters (all Miltenyi Biotec, Bergisch Gladbach, Germany) according to manufacturer’s instructions. Cells were cryopreserved and thawed following the protocol above.

### Flow cytometry

Fc Beads (BD Biosciences, NY, USA) and fluorescence minus one (FMO) were used to determine spillover values and to identify the positive and negative populations prior to analysis of patients’ samples.

Cells were washed with PBS by centrifugation (5 min at 1500*g*) and were resuspended in 100 µl 4% formalaldehyde (Cell Signaling Technology) for fixation. After 15 min, cells were washed with PBS (5 min at 1500*g*). Cells were permeabilized by adding 0.5% Tween 20 (Thermo Fisher Scientific) for 15 min, followed by a washing step. Cells were blocked with human FcR blocking reagent (BD Biosciences) for 10 min. Next, cells were incubated at RT for 15 min in the dark with PE anti-human CD68 (clone Y1/82A, BioLegend, CA, USA), BV421 anti-human CD163 (clone GHI/61, BioLegend), and Alexa Fluor 647 anti-human IDO1 antibodies (clone V50-1886, BD Biosciences).

For analysis of tumour tissue and healthy adjacent tissue, cells were additionally incubated with FITC anti-CD326 antibody (clone 9C4, BioLegend) for 15 min at RT in the dark prior to fixation.

Cells were analysed using a BD FACSVerse Flow Cytometer (BD Biosciences). FlowJo software 10.70. (Treestar, OR, USA) was used to examine cells and analyse flow cytometry data.

### Enzyme-Linked Immunosorbend Assay (ELISA)

The concentrations of Trp and Kyn were measured with commercial ELISA kits following the instructions of the manufacturer (Trp: BAE-2700, Kyn: BAE-2200, both ImmuSmol, Bordeaux, France). Optical density was measured using a microplate reader (Thermo Fisher Scientific) at a wavelength of 450 nm. Concentrations were calculated by reference to the standard curve.

### Statistical analysis

Statistical analysis was performed using the Statistical Package for the Social Sciences 27.0 (SPSS, IL, USA) and the GraphPad Prism 7.01 software.

The relationship between variables was analysed by the Spearman’s rank correlation test. The statistical comparisons between two groups were evaluated using *t* test.

One-way analysis of variance (ANOVA) followed by a multiple comparison test was used for comparison between more than two groups. Survival analysis was carried out using the Kaplan–Meier method. Log-rank test was used to determine differences between the groups. Potential prognostic factors were analysed by univariate and multivariate Cox regression.

A *p* value < 0.05 was considered statistically significant.

## Results

### Patient cohort for tissue microarrays

Immunohistochemical staining on TMAs was performed to evaluate the expression of IDO1 in PTs and LNMs of OSCC patients. A total amount of 171 PTs were investigated. Of these patients, 105 (61.4%) were male and 66 (38.6%) were female. The ages ranged from 27 to 88 years with a mean age of 64 years. The staining pattern of IDO1 was largely cytoplasmic with focal nuclear staining in some immune cells. Table S1 provides an overview of the clinicopathological characteristics of the patient cohort.

### Correlation of IDO1 expression in immune cells in primary tumours with clinicopathological characteristics

**Fig. 1:**
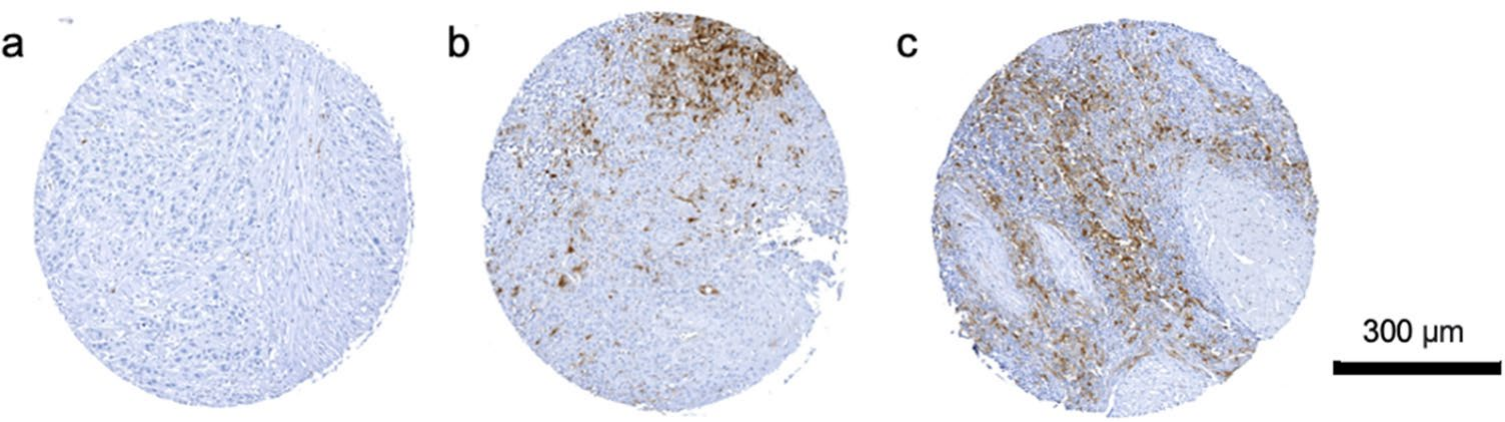
Representative images of tissue microarrays showing **a** negative, **b** moderate, and **c** strong IDO1 expression in immune cells in primary tumours of oral squamous cell carcinoma patients

High IDO1 expression in immune cells was significantly associated with female sex (SRC, *p* = 0.006), age > 75 years (SRC, *p* = 0.019), high T classification (SRC, *p* = 0.01), nodal positivity (*p* = 0.023) and high UICC stage (SRC, *p* = 0.026). Patients’ clinicopathological characteristics according to IDO1 expression in immune cells in PTs are documented in Table 1. Representative images of TMAs showing different densities of IDO1^+^ immune cells are shown in Fig. 1.

**Table 1:** Correlation of IDO1 expression in immune cells in primary tumours with clinicopathological characteristics of oral squamous cell carcinoma patients

Characteristics	Low IDO1 expression (%)	High IDO1 expression (%)	*p* value
*Sex*			
Men	49 (56.2)	46 (43.8)	0.006*
Women	23 (34.8)	43 (65.2)	
*Age*			
≤ 75	71 (52.6)	64 (47.4)	0.019*
> 75	11 (30.6)	25 (69.4)	
*T classification*			
1	32 (58.2)	23 (41.8)	0.01*
2	30 (50.8)	29 (49.2)	
3	4 (57.1)	3 (42.9)	
4	16 (32)	34 (68)	
*N classification*			
N0	63 (53.8)	54 (46.2)	0.023*
N +	19 (35.2)	35 (64.8)	
*UICC stage*			
I	28 (58.3)	20 (41.7)	0.026*
II	21 (53.8)	18 (46.2)	
III	11 (61.1)	7 (38.9)	
IV	22 (33.3)	44 (66.7)	
*Recurrence*			
Yes	70 (51.1)	67 (48.9)	0.1
No	12 (25.3)	22 (64.7)	
*Differentiation grade*			
1	7 (58.3)	5 (41.7)	0.932
2	53 (46.1)	62 (53.9)	
3	19 (50)	19 (50)	
Missing	3 (50)	3 (50)	
*PD-L1*			
Negative	23 (51.1)	22 (48.9)	0.745
Positive	54 (48.2)	58 (51.8)	
Missing	5 (35.8)	9 (64.3)	
*PD-L2*			
Negative	15 (57.7)	11 (42.3)	0.335
Positive	60 (47.2)	67 (52.8)	
Missing	7 (38.9)	11 (61.1)	
*p16*			
Negative	40 (45.4)	48 (54.5)	0.971
Positive	27 (45.8)	32 (54.2)	
Missing	15 (62.5)	9 (37.5)	

Asterisk indicates *p* value < 0.05

*UICC *Union Internationale Contre le Cancer, *PD-L1/2* programmed cell death ligand 1/2

### Survival analysis in relation to IDO1 expression in immune cells

**Fig. 2:**
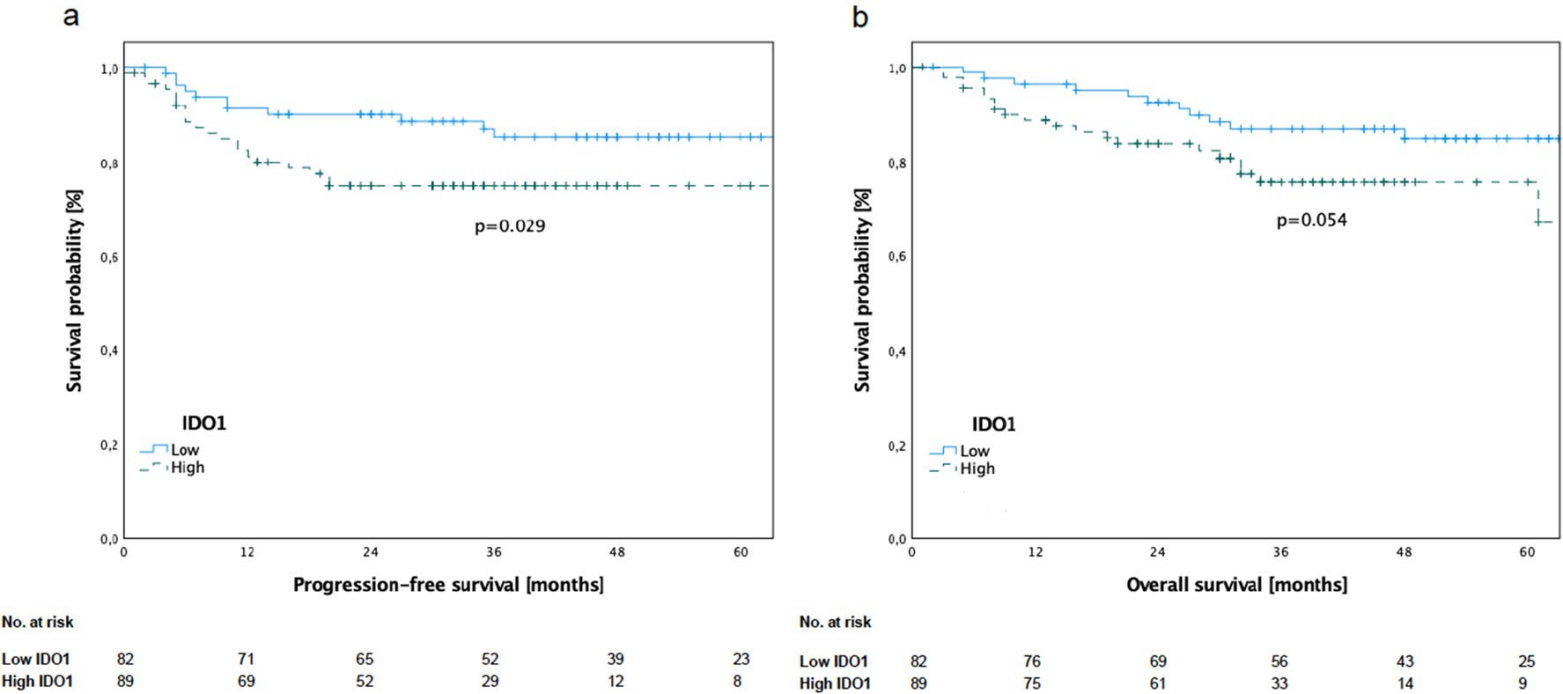
Kaplan–Meier curves of progression-free survival (PFS) and overall survival (OS) for 171 patients with OSCC according to IDO1 expression in immune cells in primary tumour. **a** Patients with high IDO1 expression showed significantly inferior PFS (*p* = 0.029). **b** Patients with high IDO1 expression showed nearly significantly inferior OS (*p* = 0.054)

The analysis revealed a significantly reduced progression-free survival (PFS; Log-rank test, *p* = 0.029) and baseline significance for worse overall survival (OS; Log-rank test, *p* = 0.054) for patients with high IDO1 expression (Fig. 2). The mean time to death was 45.44 ± 19.59 months in low IDO1 expression group, whereas it was 32.35 ± 17.69 months in the high IDO1 expression group.

To further assess the prognostic value of IDO1 expression in immune cells as well as clinicopathological characteristics, univariate and multivariate Cox regressions were performed.

Univariate Cox regression revealed the UICC stage (*p* = 0.012) and the number of IDO1^+^ immune cells (*p* = 0.029) as significant prognostic factor regarding PFS. Multivariate Cox regression confirmed the UICC stage (*p* = 0.024) and the number of IDO1^+^ immune cells (*p* = 0.046) as independent prognostic factors regarding PFS. The UICC stage was confirmed as prognostic factor in univariate (*p* = 0.004) and multivariate Cox regression (*p* = 0.012) regarding OS. Results of univariate and multivariate analyses are shown in Tables 2 and 3.

**Table 2:** Univariate analysis of clinicopathological characteristics and IDO1 expression in immune cells in primary oral squamous cell carcinomas

Characteristics	Progression-free survival		Overall survival	
	HR (95% CI)	*p* value	HR (95% CI)	*p* value
*Sex*				
Women vs. men	0.867 (0.426–1.764)	0.693	1.032 (0.501–2.127)	0.932
*Age*				
≤ 75 years vs > 75 years	1.140 (0.492–2.64)	0.601	1.644 (0.732–3.693)	0.135
*UICC stage*				
I/II vs. III/IV	2.43 (1.19–4.962)	0.012*	2.876 (1.355–6.102)	0.004*
*Immune cells*				
High vs. low IDO1 expression	1.483 (1.029–2.137)	0.029*	1.428 (0.985–2.070)	0.054

Statistically significant differences between groups were determined by Cox proportional hazard model. Asterisk indicates *p* value < 0.05

*CI *confidence interval, *HR *hazard ratio, *UICC* Union Internationale Contre le Cancer

**Table 3:** Multivariate analysis of clinicopathological characteristics and IDO1 expression in immune cells in primary oral squamous cell carcinomas

Characteristics	Progression-free survival		Overall survival	
	HR (95% CI)	*p* value	HR (95% CI)	*p* value
*Sex*				
Women vs. men	0.670 (0.233–1.393)	0.283	0.792 (0.374–1.676)	0.542
*Age*				
≤ 75 years vs > 75 years	0.978 (0.417–2.292)	0.959	1.456 (0.638–3.320)	0.372
*UICC stage*				
I/II vs. III/IV	2.296 (1.114–4.732)	0.024*	2.646 (1.237–5.662)	0.012*
*Immune cells*				
High vs. low IDO1 expression	1.469 (1.006–2.144)	0.046*	1.337 (0.910–1.965)	0.139

Statistically significant differences between groups were determined by Cox proportional hazard model. Asterisk indicates *p* value < 0.05

*CI *confidence interval, *HR *hazard ratio, *UICC *Union Internationale Contre le Cancer

### Expression of IDO1 in immune cells in lymph node metastases

Next, immunohistochemical staining on TMAs was performed to evaluate the expression of IDO1 in immune cells in LNMs of 40 OSCC patients. Representative images of TMAs showing different densities of IDO1^+^ immune cells are exemplified in Fig. S1.

IDO1^+^ immune cells were significantly associated with recurrence (SRC, *p* = 0.038, Table S2).

Patients with high IDO1 expression showed significantly inferior PFS (*p* = 0.009). No significant difference in OS was found between low and high IDO1 expression groups (*p* = 0.102) (Fig. S2).

Univariate and multivariate Cox regression revealed the number of IDO1^+^ immune cells as prognostic factor regarding PFS (univariate: *p* = 0.009; multivariate: *p* = 0.022). Results of univariate and multivariate analyses are shown in Table S3 and S4.

No significant difference was observed between IDO1 expression in immune cells between PTs and LNMs (*t* test, *p* = 0.083).

### Patient cohort for flow cytometric analysis and enzyme-linked immunosorbent assay

Tumour tissues of 9 patients and healthy adjacent tissues of 6 patients were analysed by flow cytometry. At time point of the surgery, PBMCs and serum of 19 patients were analysed by flow cytometry and ELISA. One week after surgery, blood samples of 11 patients and two weeks after surgery, blood samples of 7 patients were analysed. Furthermore, blood samples of 4 age- and gender-matched HCs were analysed. Clinicopathological characteristics of the patient cohort are shown in Table S5.

The mean age was 67 years in OSCC group and 63 years in HC group.

There were no statistically significant differences in mean age (*t* test, *p* = 0.987) and gender (*t* test, *p* = 0.522) between OSCC patients and HCs.

### Expression of CD68, CD163, and IDO1 in PBMCs

We used flow cytometry to evaluate the percentage of circulating macrophages with regard to IDO1 expression in PBMCs. Fig. S3 illustrates the gating strategy used to identify the macrophages.

**Fig. 3:**
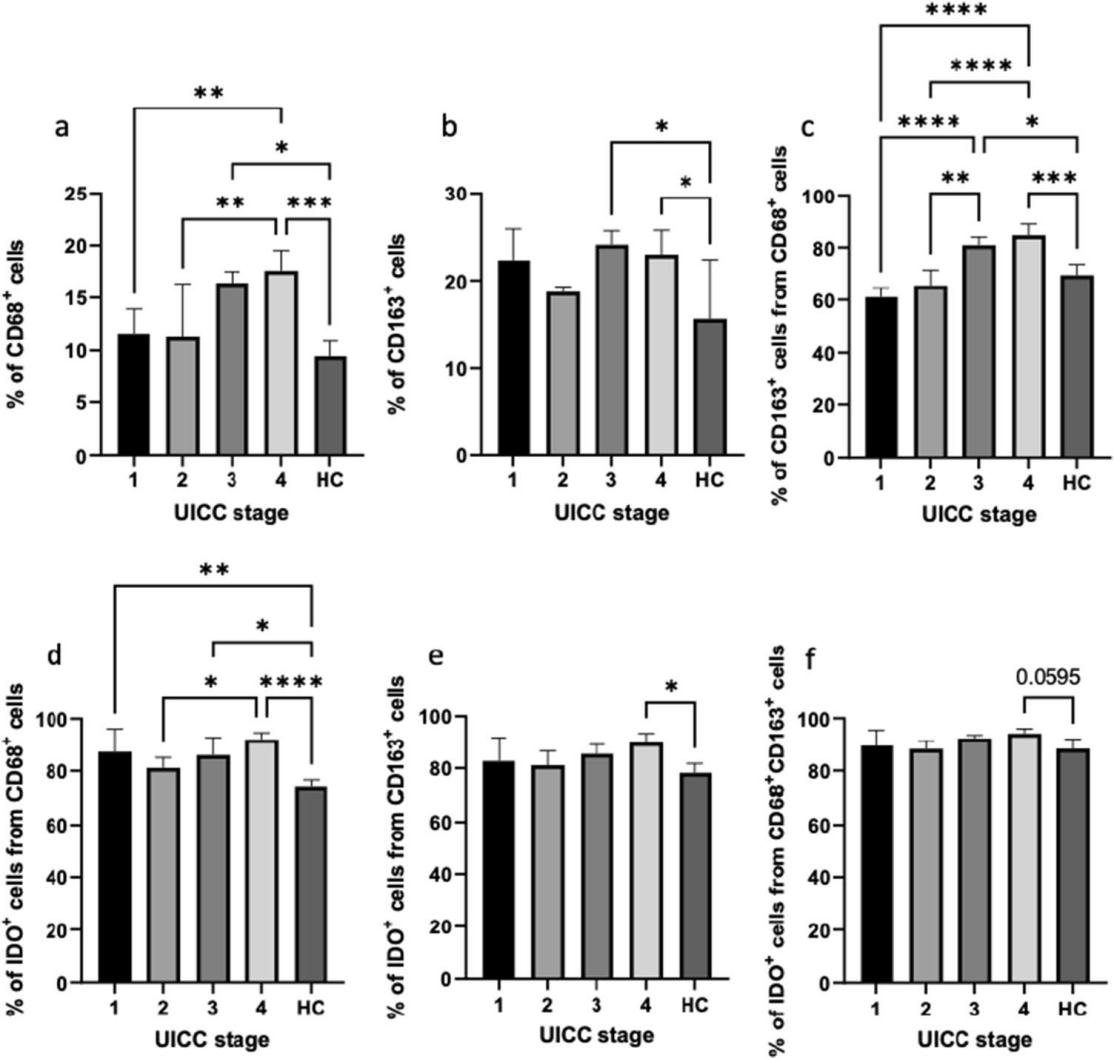
Quantification and comparison of different circulating macrophage subsets between oral squamous cell carcinoma patients and healthy control (HC) group depending on UICC stage. Graphs represents the relative number of **a** CD68^+^ cells, **b** CD163^+^ cells, **c** CD163^+^ cells gated on CD68^+^ cells, **d** IDO1^+^ cells gated on CD68^+^ cells, **(e)** IDO1^+^ cells gated on CD163^+^ cells, and **f** IDO1^+^ cells gated on CD68^+^CD163^+^ cells. *HC *healthy control, *UICC *Union Internationale Contre le Cancer. Statistical analysis was performed using one-way ANOVA, followed by a multiple comparisons test. Values are expressed as mean ± standard. Asterisks represent relevant statistical difference between groups. ∗ indicates *p* value < 0.05, ∗∗ indicates *p* value < 0.01, ∗∗∗ indicates *p* value < 0.001, and **** indicates *p* value < 0.0001

Results revealed a significant increase in circulating CD68^+^ and CD68^+^CD163^+^ macrophages in UICC stage IV compared to UICC stage I and HCs (ANOVA, *p* < 0.05). Moreover, we identified significantly higher proportions of IDO1^+^ cells among CD68^+^ and CD163^+^ macrophages in UICC stage IV compared to HCs (ANOVA, *p* < 0.05). The percentage of IDO1^+^ cells among CD68^+^CD163^+^ cells was almost significantly higher in UICC stage IV than in HCs (ANOVA, *p* = 0.0595, Fig. 3). No significant differences were found between sexes nor age groups (≤ 75 years and > 75 years).

### Distribution of TAMs in intratumoural and peritumoural areas

To examine the localisation of the TAMs, flow cytometric analyses of single cell suspensions from tumour and healthy adjacent tissues were performed.

We identified higher proportions of CD163^+^ and CD68^+^163^+^ TAMs in tumour tissue than in healthy adjacent tissue (*t* test, *p* < 0.01).

We identified a higher percentage of IDO1^+^ cells among CD68^+^ and 163^+^ TAMs in tumour tissue than in healthy adjacent tissue (*t* test, *p* < 0.01) (Fig. S4).

### Changes in IDO1 expression after tumour resection

Next, we evaluated clinical responses and quantitative changes of IDO1 in OSCC patients following tumour surgery.

We observed that IDO1 expression was increased from day 0 to day 7, followed by a significantly decrease by day 14 (*t* test, *p* = 0.01). Especially IDO1 expression in CD68^+^CD163^+^ cells was significantly reduced at 2 weeks after surgery compared to day 0 (*t* test, *p* < 0.05).

### Serum levels of Tryptophan and Kynurenine as surrogate for IDO1 activity

The serum levels of Trp and Kyn were measured via ELISA. Levels of Kyn were higher in patients with larger tumours (T3/T4) than in patients with smaller tumours (T1/T2) and HCs. However, the differences did not reach significance (ANOVA, *p* > 0.05). Levels of Trp were lower in patients with larger tumours (T3/T4) than in patients with smaller tumours (T1/T2; ANOVA, *p* > 0.05) and HCs (ANOVA, *p* < 0.05). Patients with LNMs had significantly higher levels of Kyn and significantly lower levels of Trp (*t* test, *p* < 0.05).

**Fig. 4:**
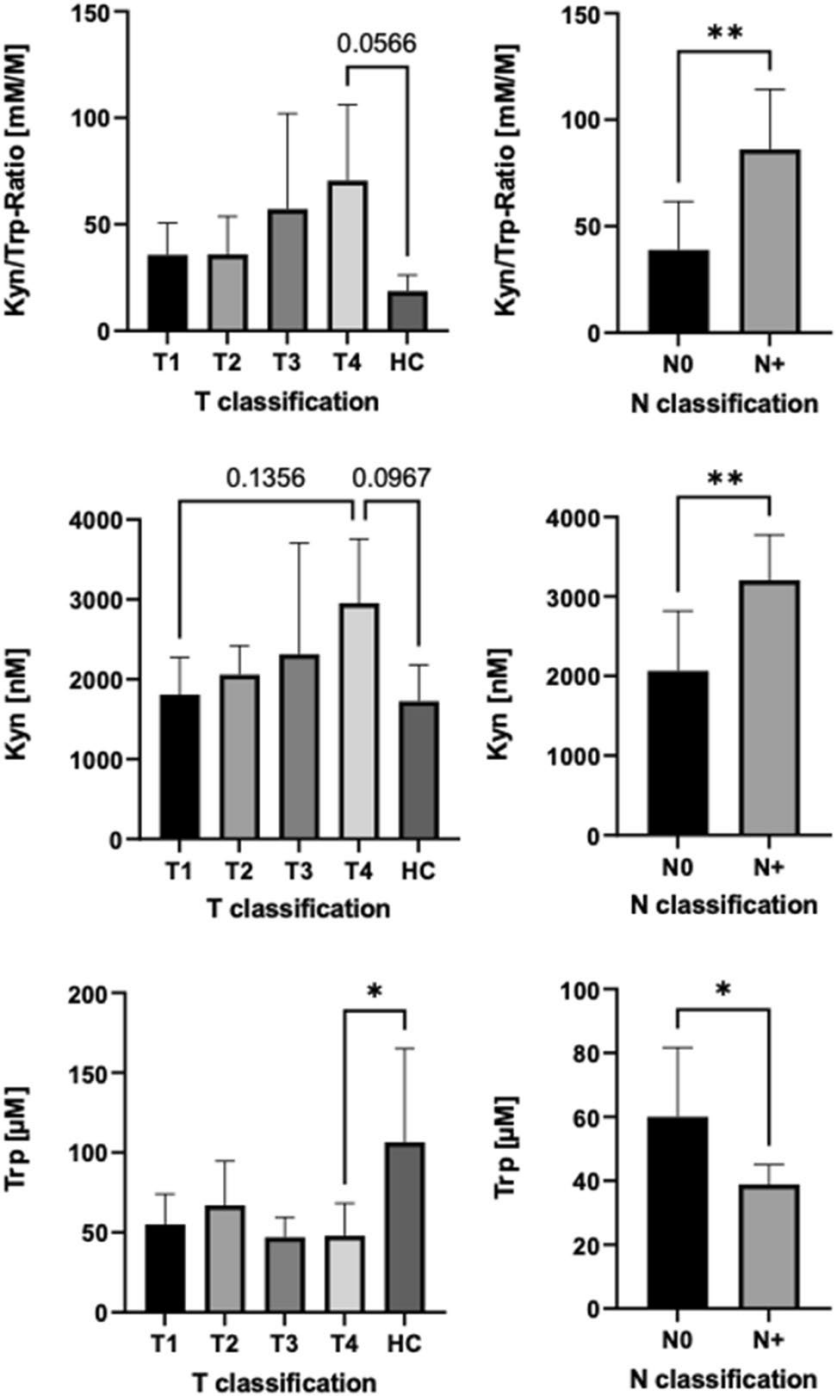
Serum concentrations of Kynurenine (Kyn), Tryptophan (Trp) and their ratio depending on T and N classification in oral squamous cell carcinoma patients. The statistical comparisons between two groups were evaluated using *t* test. One-way ANOVA followed by a multiple comparison test was used for comparisons between more than two groups. Values are expressed as mean ± standard deviation. Asterisks represent relevant statistical difference between groups. ∗ indicates *p* value < 0.05 and ∗∗ indicates *p* value < 0.01

The Kyn/Trp ratio was calculated and employed for IDO1 activity. Patients with LNMs had a significantly higher Kyn/Trp ratio than patients without LNMs (*t* test, *p* < 0.01). Moreover, patients with larger tumours (T3/T4) had a higher Kyn/Trp ratio than patients with smaller tumours (T1/T2) and HCs. However, the differences did not reach significance (ANOVA, *p* > 0.05; Fig. 4).

The Kyn/Trp ratio was correlated with IDO1 expression in PBMCs (SRC, *p* < 0.05), whereas it was not correlated with IDO1 expression in epithelial cancer cells (SRC, *p* = 0.672).

### Changes in serum levels of Tryptophan and Kynurenine after tumour resection

As shown in Fig. S5, the amount of Kyn increased in serum after tumour resection from day 0 to day 7 (*t* test, *p* > 0.05), but decreased thereafter. There was no significant difference in the amount of Kyn between day 0 compared to day 14 (*t* test, *p* = 0.385). In contrast, the amount of Trp increased from day 0 to day 7, followed by a higher increase (*t* test, *p* = 0.042). The Kyn/Trp ratio significantly increased from day 0 to day 7 and decreased by day 14. The difference between day 0 and day 14 was nearly significant (*t* test, *p* = 0.067).

## Discussion

Recent evidence indicates that macrophages in the TME contribute to immune dysregulation and therefore might play a critical role in resistance to IDO1 inhibitors. This study aims to gain insights into the significance of IDO1^+^ immune cells in OSCC patients and to further study the link between IDO1 and macrophages, which may open new avenues for developing immune-based therapies focussing on the TME. To our knowledge, this is the first study regarding the role of IDO1^+^ immune cells in OSCC patients.

In the retrospective part of this study, we investigated the IDO1 expression in immune cells in a large homogeneous cohort of OSCC patients by IHC on TMAs. In contrast to previous studies, we investigated IDO1 expression in immune cells separately from epithelial cells. Additionally, we investigated IDO1 expression in LNMs and compared it to the corresponding PTs.

We found that high IDO1 expression in immune cells in PTs was significantly associated with advanced OSCC [T classification, nodal positivity, UICC stage (SRC, *p* < 0.05)]. High IDO1 expression in immune cells in LNMs was significantly associated with recurrence (SRC, *p* = 0.038). Univariate and multivariate Cox regression revealed the number of IDO1^+^ immune cells in PTs and LNMs as significant prognostic factor regarding PFS (*p* < 0.05). No significant difference was observed between IDO1 expression in immune cells between PTs and LNMs (*t* test, *p* = 0.083).

Laimer et al. explored the expression and prognostic of IDO1 in PTs of OSCC patients by IHC and real time polymerase chain reaction. However, they did not differentiate between different cells types expressing IDO1. In contrast to our results, they could not find a significant correlation between IDO1 expression and clinical stage, age, and pathological grading. However, they reported an almost significant association between IDO1 expression and sex. Additionally, Laimer et al. found that patients with a high IDO1 expression receiving either adjuvant radio-chemotherapy or adjuvant chemotherapy alone had a significantly worse OS than patients with a low IDO1 expression (Laimer et al. 2011). On the contrary, high epithelial IDO1 expression was previously to be correlated with the number of LNMs, extranodal invasion in LNMs (Seppälä et al. 2016), and worse survival in HNSCC (Ye et al. 2013).

Results on prognostic significance of IDO1 expression in other types of cancer are heterogenous: high IDO1 expression was correlated with worse survival in gastrointestinal (Ferdinande et al. 2012; Théate et al. 2015) and gynecologic cancers (Inaba et al. 2009; Théate et al. 2015), nasopharyngeal carcinoma (Ben-Haj-Ayed et al. 2016) and melanoma (Théate et al. 2015), whereas it was correlated with better survival in renal cell carcinoma (Riesenberg et al. 2007) and hepatocellular carcinoma (Ishio et al. 2004).

TAMs are abundant in the TME and are suggested to play a crucial role in facilitating immune responses. To this point, a major obstacle remains the intricate heterogeneity of macrophages and evidence emerged that Mills paradigm of antitumour M1-like and pro-tumour M2-like macrophages has to be redefined. Results regarding the significance of TAMs in different cancers are contradictory, which might be attributed to the different cancer types but also to their functional polymorphism and distinct microenvironments. Therefore, TAM subpopulations with their specific markers and their distinct function urgently need to be further investigated (Wu et al. 2020).

Previously, macrophages were observed to be the predominant source of IDO1 expression in brain metastases of melanoma (Herrera-Rios et al. 2020). In agreement with this observation, IDO1 expression was detected in M2-macrophages in Hodgkin lymphoma and was associated with shortened survival in these patients (Choe et al. 2014).

Since we were able to detect IDO1^+^ macrophages in both, tissue samples and blood, we also investigated the presence and abundance of IDO1 expressing cells in PBMCs to estimate their suitability as a biomarker capturing for better risk stratification and therapy monitoring of OSCC patients and to assess the link between IDO1 and macrophages. We investigated CD68^+^, CD163^+^ and CD68^+^CD163^+^ cells and their IDO1^+^ percentage in PBMCs at time point of the surgery, 1 and 2 weeks after surgery prospectively.

The CD68 surface marker represents both the M1-like and M2-like subtypes of TAMs, whereas CD163 is a marker for the M2-like macrophage population (Minami et al. 2018; Ohashi et al. 2017).

We found that the frequency of total macrophages and M2-like macrophages are significantly higher in PBMCs in patients in advanced stages than in lower stages and HCs (ANOVA, *p* < 0.05). Our results coincided with the previous reports that tumours exhibiting high levels of CD68^+^ and CD163^+^ cells correlate with increased LNM (Marcus et al. 2004), extracapsular extension, and advanced stage in HNSCC (Wehrhan et al. 2014).

Moreover, Alves et al. concluded, that a high number of TAMs in the TME may be viewed as an indicator of poor prognosis (Alves et al. 2018).

Only a few studies reported on expression of IDO1 in PBMCs (Munn et al. 2002). We found that IDO1 expression overall was significantly higher in cancer patients of all stages than in HCs and also significantly higher in advanced stages than in lower stages of OSCC (ANOVA, *p* < 0.05). Moreover, IDO1 expression was significantly higher in CD68^+^ and CD163^+^ cells (ANOVA, *p* < 0.05) and almost significantly higher in CD68^+^CD163^+^ cells in UICC stage 4 than in HCs (ANOVA, *p* = 0.0595).

Altogether, our data suggest that IDO1 expressing macrophages are more abundant in blood of OSCC patients in advanced stages and might be used as a biomarker guiding decisions on IDO1 targeting therapies.

We investigated single cell suspensions of tumour tissue and healthy adjacent tissue to compare the distribution of TAM subtypes in the intra- and peritumoural area for an improved understanding of hypoxia and abnormal vasculature influencing TAMs in the TME.

We observed a significantly higher amount of CD163^+^ and CD68^+^CD163^+^ cells in the intratumoural area than in the peritumoural area. Moreover, a significantly higher proportion of CD68^+^ and CD163^+^ cells in the intratumoural area expressed IDO1 (*t* test, *p* < 0.05). TAM accumulation might be attributed to hypoxia due to the imbalance between oxygen supply and demand caused by an abnormal vasculature in the TME (Tripathi et al. 2014). Furthermore, hypoxia is suggested to hamper TAM migration (Chanmee et al. 2014).

It is well recognized that IDO1 can be induced in response to inflammation and therefore by interferon gamma (IFN-Munn et al. 1999, 2004; Taylor and Feng 1991).

However, the temporal dynamics of IDO1 activation and therefore metabolite production in human immune cells after tumour resection are currently unknown.

We observed an increase in IDO1 expressing cells from day 0 to day 7, which we attributed to the inflammatory response related to surgery. After that, IDO1 expression was significantly reduced in PBMCs (*t* test, *p* = 0.01), potentially due to the removal of the immunosuppressive tumour. Especially, IDO1 expressing CD68^+^CD163^+^ cells were significantly reduced at 2 weeks after surgery compared to day 0 (test, *p* < 0.05). These results demonstrated that IDO1 expressing immune cells are far more prevalent before resection of the PT and are changed in a time dependent manner following tumour resection. This observation is consistent with MacFarlane et al., who observed that PD-L1 expression is significantly reduced after surgical resection of the PT (MacFarlane et al. 2014).

Additionally, Economopoulou et al. investigated the significance of IDO1 mRNA levels in circulating tumour cells at baseline and after chemotherapy in HNSCC patients and found that IDO1 was significantly overexpressed at baseline compared with post-treatment (Economopoulou et al. 2020).

We quantified the Kyn and Trp levels in blood samples of OSCC patients by ELISA to define their ratio as useful serum biomarker capturing IDO1 activity. Implementing the use of non-invasive biomarkers is a relevant research target for both prognostic and therapeutic purposes to pave the way to personalized management.

In the current study, we detected an increased Kyn/Trp ratio in OSCC patients of all tumour sizes compared to HCs (ANOVA, *p* = 0.0566). Moreover, the Kyn/Trp ratio was higher in patients with T3/T4 tumours than in patients with T1/T2 tumours, but the difference did not reach significance (ANOVA, *p* > 0.05). Notably, the Kyn/Trp ratio was significantly higher in patients with LNMs (*t* test, *p* < 0.05). Therefore, it might be a suitable biomarker of metastasis in OSCC patients.

Previously, a higher Kyn/Trp ratio has been linked to higher tumour size, metastasis, and advanced disease in different solid and hematological cancer types (Brochez et al. 2017; Suzuki et al. 2010). Mandarono et al. reported that an enhanced Trp breakdown occurring in the TME, reflected by decreased Trp and elevated Kyn concentrations in the peripheral blood, is often observed and related to tumour progression and poor clinical outcome in non-small cell lung cancer patients (Mandarano et al. 2021). Moreover, increased Kynurenine levels were described to be associated with advanced tumour stages in T-cell leukaemia (Giusti et al. 1996) and colorectal carcinoma (Huang et al. 2002).

In this study, there were no significant differences in Kyn/Trp ratio, Kyn and Trp between sexes.

We quantified Kyn and Trp as key molecules of Trp metabolism and the Kyn/Trp ratio as surrogate for IDO1 activity 1 and 2 weeks after surgery. On average, the Kyn/Trp ratio was reduced after surgery. However, the difference in Kyn/Trp ratio between pre and post surgery narrowly missed significance (*p* = 0.067), which might be attributed to patient heterogeneity and different inflammatory responses to surgery.

Altogether, these data indicate a striking IDO1-mediated Trp degradation in OSCC. Moreover, our results suggest that surgery alters the IDO1-mediated immune status and that it induces significant changes in the IDO1-mediated immune activity level of cancer patients.

In our patient cohort the Kyn/Trp ratio was correlated with IDO1 expression in PBMCs, whereas it was not correlated with IDO1 expression in epithelial tumour cells. This is in contrast to an observation in human penile squamous cell carcinoma (Zhou et al. 2020), but in line with an observation in melanoma patients (Chevolet et al. 2015). We suggest that serum Kyn/Trp reflects metabolic activity of IDO1^+^ circulating immune cells.

This study has some limitations. We acknowledge that the relativly small sample size in flow cytometry and ELISA group urges its validation in larger cohorts. We observed a decrease of IDO1 expression after surgery, but investigations on IDO1 expression at later time points are necessary to investigate if IDO1 expression stays on a low level over time or increases with recurrence. In the prospective part of this study, we were not able to investigate the association between IDO1 expressing macrophages and survival rate, because the samples were collected recently.

To this day, the number of markers employed to identify populations of TAMs differs. In some studies, only a single marker is used, whereas in others, multiplex-IHC is employed. One problem of IHC might be that antigens can be colocalized in the same cell compartment and therefore interpretation might be difficult or even they colour overshadow each other. Using a technology like flow cytometry facilitated to decipher TAM subtypes by sensitively quantifying the intracellular expression of three markers of macrophages in cancer patients. Given the heterogeneity of macrophages, further studies on their subset functions are necessary.

## Conclusion

IDO1 expressing immune cells, especially macrophages, are more abundant in OSCC patients in advanced stages and are associated with reduced progression-free survival. Further investigations are needed to explore their role in local and systemic immune response. Increased insights in how the link between IDO1 and macrophages affects cancer immune escape and disease outcome may open new avenues for developing immune-based therapies. Upregulation of IDO1 in PBMCs was confirmed by the presence of enhanced Kyn/Trp ratios in serum. Higher IDO1 activity was especially associated with LNMs. Therefore, the Kyn/Trp ratio might be a suitable biomarker of metastasis in OSCC patients. Moreover, the Kyn/Trp in serum was correlated with the IDO1 expression in PBMCs and not with those in epithelial tumour cells. Hence, we suggest that the serum Kyn/Trp ratio reflects metabolic activity of IDO1 in circulating immune cells.

## Supplementary Information

Below is the link to the electronic supplementary material.

Supplementary file1 (DOCX 5292 KB)

## Data Availability

The data that support the findings of this study are available from the corresponding author upon reasonable request.
