# Supplementary material for: IDO1 is highly expressed in macrophages of patients in advanced tumour stages of oral squamous cell carcinoma
Source: J Cancer Res Clin Oncol. 2022 Aug 13;149(7):3623–35. doi: 10.1007/s00432-022-04277-7 (PMC10314853; doi:10.1007/s00432-022-04277-7)
Supplement: Supplementary file 1 — Supplementary file1 (DOCX 5292 KB) [file 432_2022_4277_MOESM1_ESM.docx]

**Table S1.** Clinicopathological characteristics of the investigated tissue microarray cohort



| **Characteristics** | **Number of patients (%)** |
| --- | --- |
| **Sex** |  |
| **Men** | 105 (61.4) |
| **Women** | 66 (38.6) |
| **Age** |  |
| **≤ 75 y** | 135 (78.9) |
| **> 75 y** | 36 (21.1) |
| **T classification** |  |
| **1** | 55 (32.2) |
| **2** | 59 (34.5) |
| **3** | 7 (4.1) |
| **4** | 50 (29.2) |
| **N classification** |  |
| **N0** | 117 (68.4) |
| **N+** | 54 (31.6) |
| **M classification** |  |
| **0** | 171 (100) |
| **1** | 0 (0) |
| **UICC stage** |  |
| **I** | 48 (28.1) |
| **II** | 39 (22.8) |
| **III** | 18 (10.5) |
| **IV** | 66 (38.6) |
| **Differentiation grade** |  |
| **1** | 12 (7.0) |
| **2** | 115 (67.3) |
| **3** | 38 (22.2) |
| **Missing** | 6 (3.5) |
| **Recurrence** |  |
| **Yes** | 34 (19.9) |
| **No** | 137 (80.1) |
| **P16** |  |
| **Positive** | 59 (34.5) |
| **Negative** | 88 (51.5) |
| **Missing** | 24 (14) |
| **HPV-DNA** |  |
| **Negative** | 154 (90.1) |
| **Missing** | 17 (9.9) |

Abbreviations: DNA = desoxyribonucleic acid, HPV = human papillomavirus, UICC = Union Internationale Contre le Cancer

**Table S2.** Correlation of IDO1 expression in immune cells in lymph node metastases with clinicopathological characteristics of oral squamous cell carcinoma patients

| **Characteristics** | | Low IDO1 expression (%) | High IDO1 expression (%) | P value |
| --- | --- | --- | --- | --- |
| Sex | **Men** | 18 (64.3) | 10 (35.7) | 0.079 |
|  | **Women** | 11 (91.7) | 1 (8.3) |  |
| Age | **≤ 75** | 24 (72.7) | 9 (27.3) | 0.946 |
|  | **> 75** | 5 (71.4) | 2 (28.6) |  |
| T classification | **1** | 5 (83.3) | 1 (16.7) | 0.477 |
|  | **2** | 11 (73.3) | 4 (26.7) |  |
|  | **3** | 3 (75) | 1 (25) |  |
|  | **4** | 10 (66.6) | 5 (33.3) |  |
| N classification | **1** | 9 (69.2) | 4 (30.8) | 0.387 |
|  | **2b** | 12 (66.7) | 6 (33.3) |  |
|  | **2c** | 7 (87.5) | 1 (12.5) |  |
|  | **3** | 1 (100) | 0 (0) |  |
| UICC stage | **III** | 7 (77.8) | 2 (22.2) | 0.696 |
|  | **IV** | 22 (71) | 9 (29) |  |
| Recurrence | **No** | 23 (82.1) | 5 (17.9) | 0.038* |
|  | **Yes** | 6 (50) | 6 (50) |  |
| Differentiation grade | **2** | 19 (65.5) | 10 (34.5) | 0.145 |
|  | **3** | 9 (90) | 1 (10) |  |
|  | **Missing** | 1 (100) | 0 (0) |  |
| **PD-L1** | **Negative** | 4 (80) | 1 (20) | 0.587 |
|  | **Positiv** | 12 (66.7) | 6 (33.3) |  |
|  | **Missing** | 13 (76.5) | 4 (23.5) |  |
| **PDL-2** | **Negative** | 1 (100) | 0 (0) | 0.521 |
|  | **Positive** | 15 (68.2) | 7 (31.8) |  |
|  | **Missing** | 13 (76.5) | 4 (23.5) |  |
| **p16** | **Negative** | 11 (84.6) | 2 (15.4) | 0.199 |
|  | **Positive** | 6 (60) | 4 (40) |  |
|  | **Missing** | 12 (70.6) | 5 (29.4) |  |

Asterisk indicates p value <0.05.

Abbreviations: DNA = desoxyribonucleic acid, PD-L1/2 = Programmed death ligand 1/2, UICC = Union Internationale Contre le Cancer

**Table S3.** Univariate analysis of clinicopathological characteristics and IDO1 expression in lymph node metastases of oral squamous cell carcinoma patients

| **Characteristics** | | **Progression-free survival** | | **Overall survival** | |
| --- | --- | --- | --- | --- | --- |
|  |  | **HR (95% CI)** | **P value** | **HR (95% CI)** | **P value** |
| Sex | Women vs. men | 0.605  (0.195-1.88) | 0.374 | 0.39  (0.112-1.358) | 0.121 |
| Age | ≤ 75 years vs.  > 75 years | 2.646  (1.301-10.219) | 0.057 | 2.322  (0.726-7.423) | 0.139 |
| UICC stage | III vs. IV | 2.531  (0.574-11.166) | 0.199 | 2.837  (0.646-12.455) | 0.144 |
| Immune cells | High vs. low IDO1 expression | 1.859  (1.127-3.066) | 0.009* | 1.503  (0.905-2.495) | 0.102 |

Statistically significant differences between groups were determined by Cox proportional hazard model. Asterisk indicates p value <0.05.

Abbreviations: CI = confidence interval, HR = hazard ratio, UICC = Union Internationale Contre le Cancer

**Table S4.** Multivariate analysis of clinicopathological characteristics and IDO1 expression in lymph node metastases of oral squamous cell carcinoma patients

| **Characteristics** | | **Progression-free survival** | | **Overall survival** | |
| --- | --- | --- | --- | --- | --- |
|  |  | **HR (95% CI)** | **P value** | **HR (95% CI)** | **P value** |
| Sex | Women vs. men | 0.662 (0.179-2.448) | 0.537 | 0.358 (0.088-1.447) | 0.149 |
| Age | ≤ 75 years vs. > 75 years | 5.355 (1.619-17.058) | 0.006* | 3.188 (0.889-11.438) | 0.075 |
| UICC stage | III vs. IV | 1.133 (0.229-5.606) | 0.878 | 2.159 ( 0.484-9.638) | 0.313 |
| Immune cells | High vs. low IDO1 expression | 1.975 (1.102-3.538) | 0.022* | 1.253 (0.731-2.147) | 0.412 |

Statistically significant differences between groups were determined by Cox proportional hazard model. Asterisk indicates p value <0.05.

Abbreviations: CI = confidence interval, HR = hazard ratio, UICC = Union Internationale Contre le Cancer

**Table S5.** Descriptive data regarding demographic and clinicopathological characteristics of the prospective cohort of oral squamous cell carcinoma patients and healthy controls

| **Characteristics** | **Number of OSCC patients (%)** | **Number of patients in healthy control group (%)** |
| --- | --- | --- |
| **Sex** |  |  |
| **Men** | 9 (47.4) | 2 (50) |
| **Women** | 10 (52.6) | 2 (50) |
| **Age** |  |  |
| **≤75 y** | 14 (73.7) | 3 (75) |
| **>75 y** | 5 (26.3) | 1 (25) |
| **T classification** |  |  |
| **1** | 4 (21.1) |  |
| **2** | 4 (21.2) |  |
| **3** | 3 (15.8) |  |
| **4** | 8 (41.1) |  |
| **N classification** |  |  |
| **N0** | 14 (73.7) |  |
| **N+** | 5 (26.3) |  |
| **M classification** |  |  |
| **0** | 19 (100) |  |
| **1** | 0 (0) |  |
| **UICC stage** |  |  |
| **I** | 4 (21.1) |  |
| **II** | 3 (15.8) |  |
| **III** | 3 (15.8) |  |
| **IV** | 9 (47.4) |  |
| **Differentiation grade** |  |  |
| **1** | 2 (10.5) |  |
| **2** | 6 (31.6) |  |
| **3** | 8 (42.1) |  |
| **Missing** | 3 (15.8) |  |

Abbreviation: UICC = Union Internationale Contre le Cancer


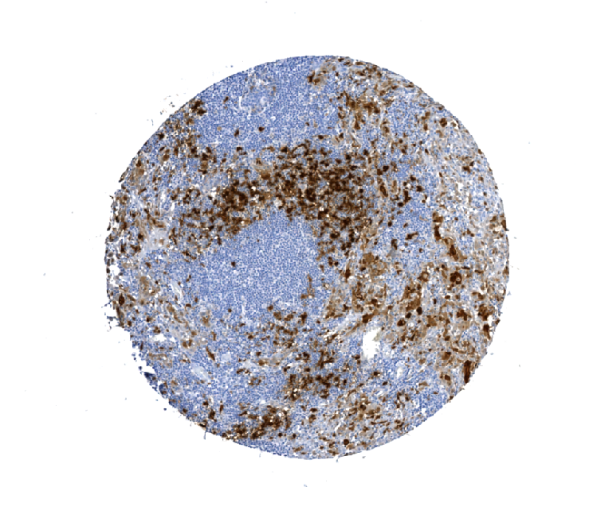
**
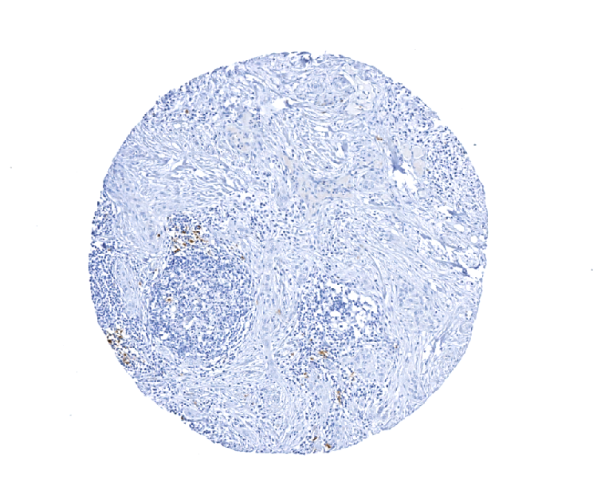

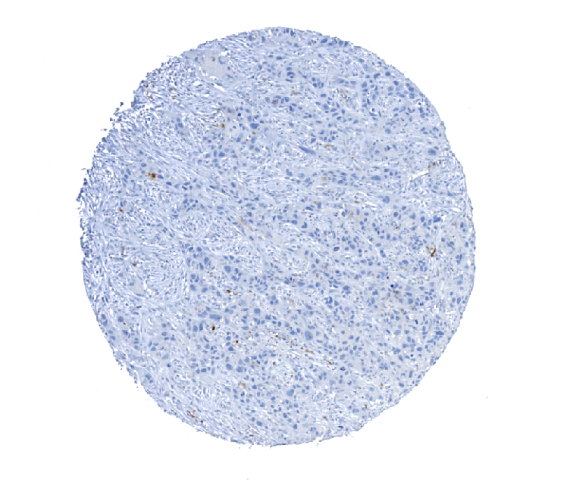
**

c

b

a

300 µm

**Fig. S1** Representative images of tissue microarrays showing **(a)** negative, **(b)** moderate, and **(c)** strong IDO1 expression in immune cells in lymph node metastases of oral squamous cell carcinoma patients

| **No. at risk** |  |  |  |  |  |  |
| --- | --- | --- | --- | --- | --- | --- |
| **Low IDO1** | 29 | 19 | 15 | 9 | 4 | 1 |
| **High IDO1** | 11 | 3 | 2 | 1 | 1 | 0 |


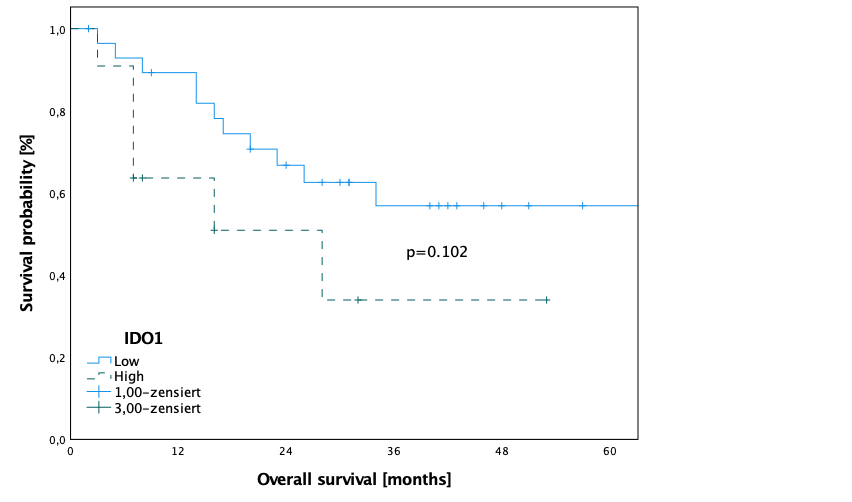

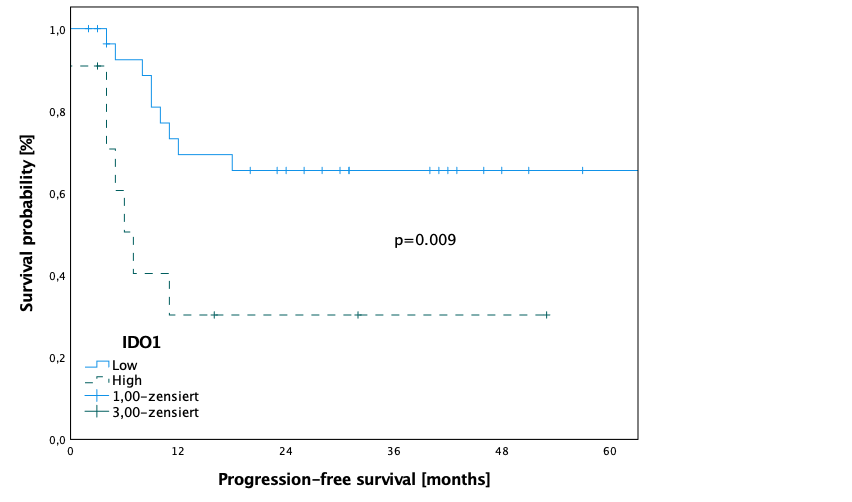


b

a


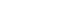

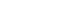


B

A


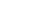

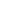

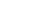

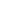

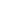

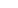

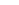

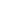

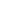

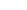

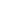

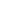

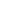

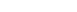

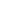

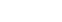

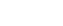


| **No. at risk** |  |  |  |  |  |  |
| --- | --- | --- | --- | --- | --- | --- |
| **Low IDO1** | 29 | 24 | 17 | 10 | 5 | 2 |
| **High IDO1** | 11 | 5 | 3 | 1 | 1 | 0 |

**Fig. S2** Kaplan-Meier curves of progression-free survival (PFS) and overall survival (OS) for 40 patients with lymph node metastases occuring in OSCC. **(a)** Patients with high IDO1 expression in immune cells showed significantly inferior PFS (p=0.009.). **(b)** No significant difference in OS was noted between low and high IDO1 expression in immune cells (p=0.102)


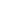

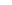

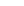

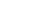

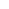

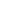

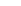

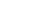

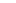

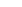

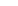

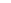

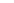

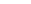

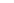

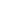

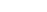

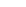

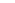

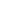

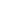

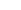

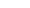


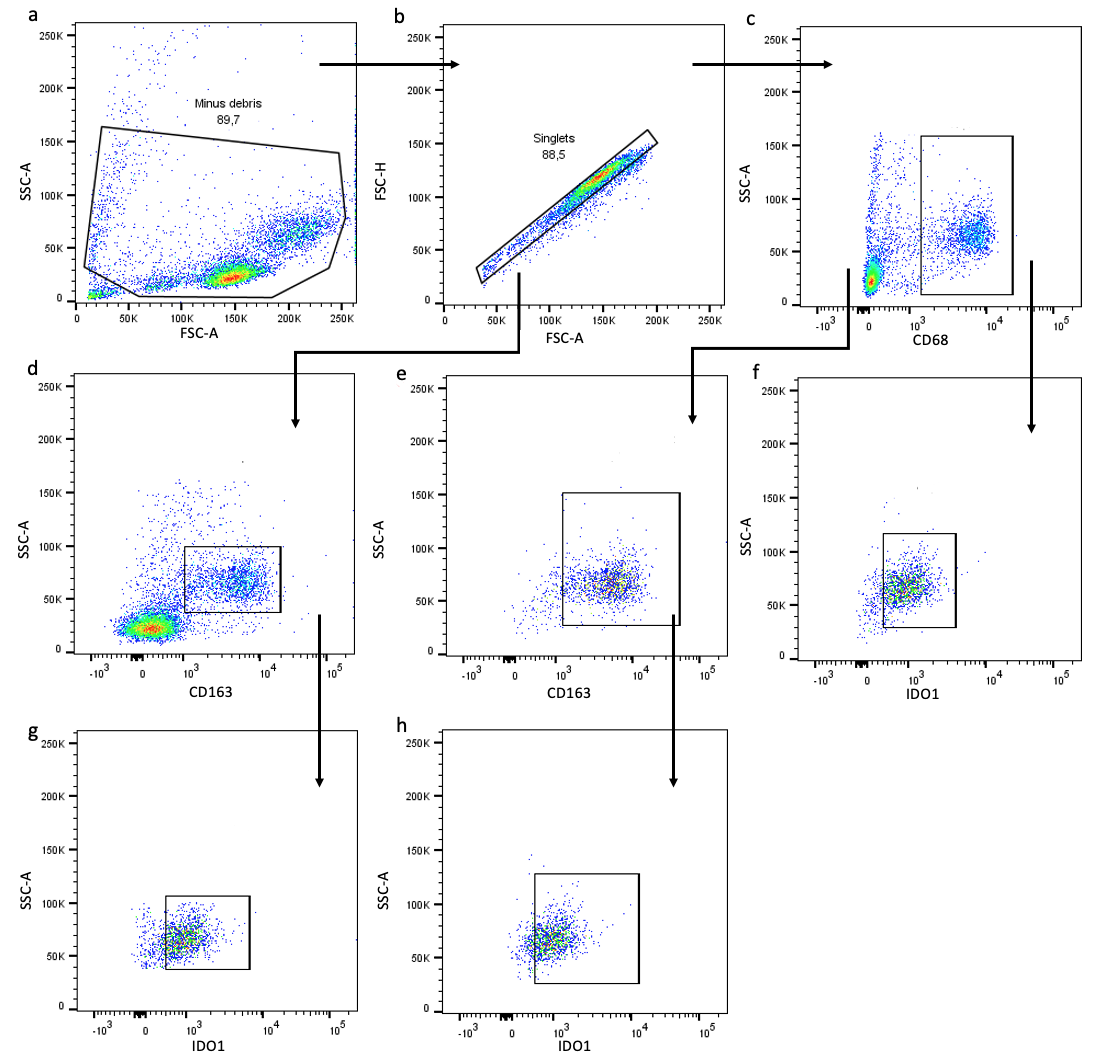


**Fig. S3** Flow cytometric analysis of CD68, CD163, and IDO1 expression on macrophages.

**(a)** SSC-A and FSC-A were used as parameters to exclude cellular debris from all recorded events. **(b)** Single cells were gated using FSC-A and FSC-H, thereby excluding doublets. **(c)** CD68^+^ cells were defined as macrophages. **(d)** CD163^+^ cells were gated. **(e)** From the CD68 gate, the expression of CD163 was assessed. The percentage of IDO1^+^ cells was investigated from the **(f)** CD68^+^ cells, **(g)** CD163^+^ cells, and **(h)** CD68^+^CD163^+^ cells


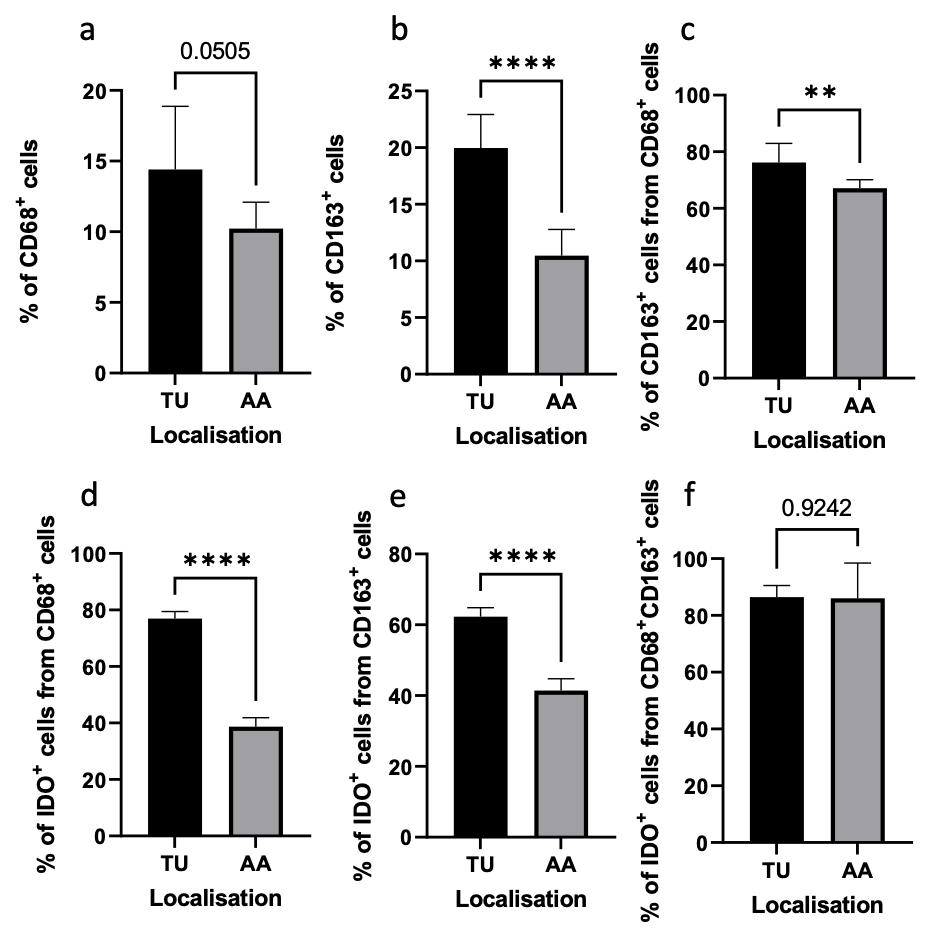


**Fig. S4** Quantification and comparison of different macrophage subsets between tumour tissue (TU) and adjacent area (AA) in oral squamous cell carcinoma patients.

Graphs represents the relative number of **(A)** CD68^+^ cells, **(B)** CD163^+^ cells, **(C)** CD163^+^ cells gated on CD68^+^ cells, **(D)** IDO1^+^ cells gated on CD68^+^ cells, **(E)** IDO1^+^ cells gated on CD163^+^ cells, and **(F)** IDO1^+^ cells gated on CD68^+^CD163^+^ cells.

The statistical comparisons were evaluated using t-test.

Values are expressed as mean ± standard deviation. Asterisks represent relevant statistical difference between groups. ^∗∗^ indicates p value <0.01 and **** indicates p value < 0.0001

**
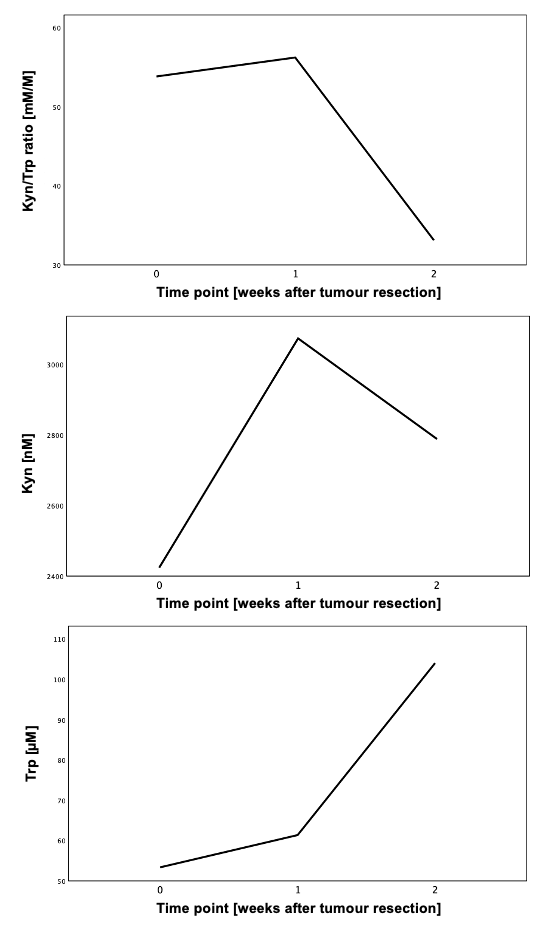
**

**Fig. S5** Serum concentrations of Kynurenine (Kyn), Tryptophan (Trp) and their ratio over time during the first two weeks after tumour resection in oral squamous cell carcinoma patients
